# Bioactive Peptides from *Astrocaryum murumuru* Residual Biomass: Enzymatic Hydrolysis, Characterization, and Functional Activities

**DOI:** 10.3390/foods15112002

**Published:** 2026-06-04

**Authors:** Gabriela Vieira Pantoja, Josué Manoel Souza Serrão, Vinicius Sidônio Vale Moraes, José Aparecido Ferreira de Lima, Emídio Beraldo-Neto, Johnatt Allan Rocha de Oliveira, Luiza Helena da Silva Martins, Daniel Carvalho Pimenta, Gustavo Guadagnucci Fontanari

**Affiliations:** 1 Programa de Pós-Graduação em Ciência e Tecnologia de Alimentos (PPGCTA), Universidade Federal do Pará (UFPA), Rua Augusto Corrêa, Belém 66075-900, PA, Brazil; gabivieirapantoja@gmail.com (G.V.P.); viniciusmoraes31@gmail.com (V.S.V.M.); 2 Instituto de Saúde e Produção Animal (ISPA), Universidade Federal Rural da Amazônia (UFRA), Belém 66077-830, PA, Brazil; jserrao362@gmail.com (J.M.S.S.); luiza.martins@ufra.edu.br (L.H.d.S.M.); gustavo.fontanari@ufra.edu.br (G.G.F.); 3 Laboratório de Bioquímica do Instituto Butantan, São Paulo 05503-900, SP, Brazil; jose.aparecido@fundacaobutantan.org.br (J.A.F.d.L.); emidio.beraldo@butantan.gov.br (E.B.-N.); 4 Faculdade de Nutrição (FANUT), Instituto de Ciências da Saúde (ICS), Universidade Federal do Pará (UFPA), Rua Augusto Corrêa, Belém 66075-900, PA, Brazil; johnatt@ufpa.br; 5 Laboratório de Ecologia e Evolução, Instituto Butantan, São Paulo 05503-900, SP, Brazil

**Keywords:** bioactive peptides, antimicrobial activity, waste valorization, protein hydrolysates, *A. murumuru*

## Abstract

*Astrocaryum murumuru* Mart., an Amazonian oilseed, generates large amounts of protein-rich residual biomass after oil extraction, representing an untapped biotechnological resource. This study aimed to obtain peptide-rich hydrolysates from this biomass via enzymatic hydrolysis (Alcalase and trypsin) and evaluate their antioxidant and antimicrobial activities. Peptides were successfully generated and characterized by RP-HPLC and MALDI-TOF (*m*/*z* ~ 500–5000). Significant functional differentiation between enzymes was observed: Alcalase hydrolysate exhibited the highest DPPH antioxidant capacity (49.82 µmol TE/g), markedly surpassing the trypsin hydrolysate and protein concentrate. Conversely, trypsin hydrolysate displayed potent and selective antimicrobial activity against *Staphylococcus aureus*, achieving near-complete growth inhibition (99.85%) at 5 mg/mL, with both hydrolysates showing strong growth inhibition (>92%) at 2.5 mg/mL. This is the first work that reports the use of an *A. murumuru* biomass to obtain bioactive peptides, and its results demonstrated that this biomass can be better explored and be an option for scientific and technological bioeconomy, generating bioinputs with sustainable applications in the pharmaceutical and food industries.

## 1. Introduction

The Amazon rainforest is rich in biodiversity, particularly in terms of its plant life. Many of these possess pharmacological and nutraceutical properties, primarily due to the presence of bioactive compounds [1].

*Astrocaryum murumuru* is an Amazonian oilseed palm that is part of the production chain of non-timber forest products, predominantly exploited by extractive producers organized in associations and cooperatives [2]. Its fruits provide inputs for the food and cosmetic industries, while its leaves, in addition to their traditional use in roofing, provide fibers for making handicrafts. The lipid fraction extracted from its kernel, commercially known as murumuru butter due to its solid consistency at room temperature, is widely used in the formulation of cosmetics, such as soaps, creams, and shampoos [3]. In the food industry, this butter acts as a partial substitute for cocoa butter, providing greater thermal stability and firmness to chocolates marketed in warm climate regions.

Lipid extraction is the main economic use of this oilseed, a process that generates a high volume of residual biomass, which comprises a series of compounds with high biological activity and contains proteins, nitrogenous compounds, and minerals [4]. When these biomasses are not used as fertilizer or silage for animal feed, they are generally unusable and improperly discarded in the environment, causing the accumulation of solid waste that may cause health problems as they are vectors for insects and rodents that transmit diseases, in addition to contributing to the increase in the greenhouse effect on the planet [5]. The sustainable harvesting and use of these oilseeds support the local economies of Amazonian communities and contribute to the conservation of the rainforest ecosystem, promoting biodiversity-friendly practices [6].

Previous studies have described that there are bioactive compounds in *A. murumuru*, mainly phenolic compounds, including gallic acid and ellagic acid, known for sequestering free radicals and preventing cellular damage [7,8]. The seed also contains abundant flavonoids, such as quercetin and kaempferol, which contribute to its anti-inflammatory and anticancer properties and promote cardiovascular health [9].

Waste reuse is a promising strategy to help address the challenges posed by climate change and environmental pollution, which have been a concern to humanity and large government agencies. In this context, the use of oleaginous biomass is interesting since, according to [10], the burning of biomass is the second leading cause of greenhouse gas emissions, contributing 8% to 10% of emissions in the Brazilian Amazon region.

The biotechnological exploration of this agro-industrial residue for obtaining and bioprospecting peptides adds value to the fruit’s production chain, encouraging the preservation of the standing forest instead of logging. In addition to boosting the local bioeconomy, this approach establishes new perspectives for proteomic analysis and the technological application of residual proteins from murumuru.

A promising approach for utilizing oleaginous biomass from the Amazon is its use for extracting proteins, which can subsequently be hydrolyzed to obtain bioactive peptides.

Bioactive peptides are molecules that present a wide range of functionalities, including antimicrobial, antioxidant, and anti-inflammatory activities, and even therapeutic properties, such as modulating the immune system and promoting wound healing [11,12,13]. These characteristics make these compounds extremely valuable for the pharmaceutical, cosmetic, and food industries.

Peptides are obtained through chemical or enzymatic hydrolysis of extracted proteins, a subtle and safe process since the working conditions are mild. The enzymes will act specifically on that substrate without issues with reactor corrosion, separation column problems, or amino acid destruction, as occurs in chemical hydrolysis processes [14]. Peptidases used for enzymatic hydrolysis are most commonly Alcalase or trypsin.

According to [15], implementing a bioeconomy based on waste biomass in the Amazon faces several technical, economic, and social challenges. These include the need to advance the knowledge and infrastructure necessary to promote the development of the bioeconomy in the region, ensure that the use of waste cakes is done in a socially fair manner, align the use of this waste with the interests of the community that produces the seeds, and be ecologically sustainable.

Oilseed waste is sustainable because it avoids wastage and adds value to byproducts through responsible resource use. This study is aligned with sustainable development goals—SDGs 12 (Responsible Consumption and Production), 9 (Industry, Innovation, and Infrastructure), and 13 (Climate Action). It is aligned with increased sustainable practices and technologies with reduced environmental impacts [16]. Additionally, it strengthens the Amazon bioeconomy, generating opportunities and contributing to forest conservation efforts.

In this context, this work aimed to utilize *A. murumuru* Mart. biomass as a source of bioactive peptides. We have assessed the hydrolysate antioxidant and antimicrobial properties. There are few studies that have been conducted on the extraction of phenolic compounds from these residues, and there are no reports in the literature to the best of our knowledge that have analyzed peptides from this biomass, making this a pioneering study in this field.

## 2. Materials and Methods

### 2.1. Sample Collection

*Astrocaryum murumuru* biomass was supplied by AmazonOil (Ananindeua, PA, Brazil). The biomass was triturated in a hammer grinder using a 0.4 mm sieve, defatted with n-hexane (1:6 *w*/*v*) for 6 h under constant shaking, and oven-dried at 50 °C to remove the solvent residues. The resulting defatted *A. murumuru* biomass was then homogenized, packed in polyethylene bags, and stored at −18 °C until analysis. All the chemicals and reagents used in this work were of analytical and HPLC grade. The enzyme Alcalase^®^ produced by *Bacillus licheniformis* (activity 2.984 U/mL), also known as subtilisin, was obtained from Merck^®^ (Burlington MA, USA), and trypsin (activity ≥ 10,000 BAEE units/mg) was obtained from Sigma-Aldrich (St. Louis, MO, USA). The use of this plant residue is registered in the National System for the Management of Genetic Heritage and Associated Traditional Knowledge (SisGen) under the number A2D84C7.

### 2.2. Determination of Centesimal Composition

The centesimal composition determinations carried out included moisture (n. 925.09), protein (n. 920.87), ash (n. 923.03), and lipids (n. 920.85), all of which were performed by the AOAC methods [17] on a dry basis. The acid and neutral detergent fiber content was determined by difference and compared with the literature [18].

### 2.3. Physicochemical Effects on Protein Solubility

These analyses were carried out following the method developed by [19], with modifications, and were based on the evaluation of the influence of the pH and ionic strength of the solute on protein solubility. Briefly, 1 g defatted biomass was placed in 20 mL of distilled water, and the pH was adjusted within a range of 1–12 using 0.5 mol/L HCl and/or NaOH. The material was stirred for 1 h at 25 °C, then centrifuged at 10,000 rpm for 20 min at 4 °C (3K30, Sigma, Osterode am Harz, Germany), and the soluble nitrogen was determined in the supernatant by the Kjeldahl method. The combined effect of NaCl concentration and pH variation on protein extraction was evaluated. The samples were dispersed in a 0.2 mol/L NaCl solution, following the same procedure mentioned above.

#### 2.3.1. Protein Extraction from Biomass

The biomass proteins were extracted following a conventional method for obtaining protein isolates by alkaline solubilization and isoelectric precipitation, as reported in previous studies from our group [20,21], with mild modifications in the extraction step. The biomass was extracted under two different weight/volume ratio conditions (in a 1:10 and 1:20 *w*/*v* proportion) in distilled water with an adjustment at conditions of pH 10 and 11. The suspension was stirred for 60 min at room temperature (25 °C ± 5) and then centrifuged at 10,000× *g* for 20 min, under 4 °C using a centrifuge 3K30, Sigma, Osterode am Harz, Germany. Afterwards, the supernatant was filtered through filtered paper and adjusted with HCl 0.1 mol/L to pH 4.5 for the isoelectric precipitation (pI) of protein and then centrifuged at 10,000× *g* for 20 min under the same conditions cited above. The protein pellet was re-suspended in water, adjusted to pH 7.0 with NaOH 0.5 mol/L, freeze-dried, and homogenized. The total nitrogen was determined by the Kjeldahl method, and the protein was calculated using the conversion factor of 6.25.

#### 2.3.2. SDS-PAGE Electrophoresis

One-dimensional SDS-PAGE electrophoresis was performed according to the methodology of [22], where 10 mg of precipitated protein sample of *A. murumuru* was solubilized in 0.5 mL buffer (Tris-HCl 0.2 M pH 6.8, SDS 40% (*v*/*v*)), 0.1% bromophenol blue and β-mercaptoethanol 20% (*v*/*v*), heated to 100 °C for 2 min and then subjected to a 12% SDS-PAGE gel, 7 cm, 1.0 mm, at 30 V for 30 min and 100 V until the end of the run. The gels were stained with Coomassie blue (GE Healthcare, Chicago, IL, USA) according to the manufacturer’s instructions to obtain the images.

### 2.4. Enzymatic Hydrolysis

The hydrolyses were performed on the protein extracted from the biomass according to the methodology already used by the group in previous studies [23], with modifications.

For Alcalase hydrolysis, suspensions containing 2% (*w*/*v*) of the extracted protein were digested for 3 h in a 50 mM sodium phosphate buffer, pH 7.6, with 1:100 enzyme:substrate, *Bacillus licheniformis*, at 50 °C. Hydrolysis was stopped by heating at 80 °C for 20 min, then cooling in an ice bath for 15 min.

For trypsin hydrolysis, suspensions containing 2% (*m*/*v*) of the extracted protein were digested for 17 h in a 50 mM sodium phosphate buffer solution containing the enzyme trypsin (Merck^®^, activity (activity ≥ 10,000 BAEE units/mg) in an enzyme/substrate ratio of 1:100 (*v*/*m*) at pH 7.0 and temperature of 37 °C. Hydrolysis was stopped by heating at 80 °C for 20 min, then cooling in an ice bath for 15 min.

Both hydrolysates were centrifuged at 10,000× *g* for 20 min at 4 °C using a centrifuge 3K30, Sigma, Osterode am Harz, Germany, and the supernatants were collected and lyophilized. The content of soluble protein of the hydrolysates was determined by the Lowry et al. [24] method using bovine serum albumin as the standard (OmniPur BSA, Fraction V, Cold Alcohol Isolation, Sigma Aldrich, St. Louis, MS, USA).

### 2.5. Characterization of Hydrolysates

A liquid chromatographer (Shimadzu Co, Kyoto, Japan) was used with an LC20AT quaternary pump and a degasser DGU-20A5R, coupled to a Rheodyne manual injector and detector UV/vis photodiode array SPD-M20A (Shimadzu Co, Kyoto, Japan). The detection wavelength was set to 220 nm, and spectra of each peak were also recorded in the range of 190–400 nm. The chromatographic separation was performed on a 250 × 4.6 mm C18 reversed-phase Gemini column, a particle size of 5 μm, and a pore size of 110 Å (Phenomenex, CA, USA). The mobile phase gradient that was set with solvent A was 0.045% trifluoroacetic acid (TFA) in water, and solvent B was 0.0365% TFA (*v*/*v*) in acetonitrile. The sample was eluted with a gradient of 5 to 95% buffer B for 30 min, at a 1 mL/min constant flow rate.

### 2.6. Matrix-Assisted Laser Desorption/Ionization Time-of-Flight (MALDI-TOF) Mass Spectrometry

Mass spectra of the protein hydrolysates were acquired using a MALDI-TOF mass spectrometer (AXIMA Performance, Shimadzu Co., Kyoto, Japan). Prior to analysis, samples were mixed in a 1:1 (*v*/*v*) ratio with a saturated matrix solution of α-cyano-4-hydroxycinnamic acid. Aliquots of this mixture (0.4–0.8 µL) were spotted onto the sample plate and allowed to air-dry. Analyses were performed in positive linear mode over a mass range of 0.1 to 25 kDa. Instrument control and data acquisition were managed using the Launchpad software version 2.9.3 (Shimadzu Biotech, Kyoto, Japan).

### 2.7. Total Phenolic Content (TPC)

The TPC was determined using the methodology of Singleton et al. [25], with adaptation for microplate analysis; the absorbance was measured at 765 nm using a microplate spectrophotometer (Multiskan Sky, Thermo Scientific, Go-SN-1530-8001397, Vantaa, Finland). Quantification was performed using gallic acid monohydrate (98%, Neon, São Paulo, Brazil) as the standard at concentrations ranging from 5 to 200 mg/L to construct the analytical curve. Based on the straight-line equation (y = 0.0069x + 0.93, R^2^ = 0.9899), the content was initially calculated in mg/mL. Subsequently, this value was converted considering the mass of the extract and expressed in milligrams of gallic acid equivalent per gram of dry extract (mg GAE/g dry extract). The detection limit (DL) was 58.83 mg/mL, and the quantification limit (LQ) was 178.26 mg/mL with a standard deviation (σ) of 0.123.

### 2.8. Antioxidant Activity (AA) by ABTS

The AA of the samples against the free radical ABTS^•+^ (2,2′-azino-bis (3-ethylbenzothiazoline-6-sulfonic acid)) was determined using the methodology of Rufino et al. [26]. Quantification was performed using Trolox ((±)-6-hydroxy-2,5,7,8-tetramethylchroman-2-carboxylic acid, 97%, Sigma, Cotia, SP, Brazil), and the results are expressed as µM of Trolox equivalent per gram of extract (µM TE/g of sample) based on the straight-line equation (y = −0.0002x + 0.7055; R^2^ = 0.9902). The detection limit (DL) was 165 µM/mL, and the quantification limit (LQ) was 500 µM/mL, with a standard deviation (σ) of 0.01.

### 2.9. AA by DPPH

The quantification of AA was performed using the DPPH method described by Brand-Williams et al. [27], with modifications; the absorbance was measured by a microplate spectrophotometer (Multiskan Sky, Thermo Scientific, Go-SN-1530-8001397, Vantaa, Finland) at a wavelength of 520 nm. The quantification of the results was performed by interpolating the absorbance of the samples against a calibration curve constructed with standard concentrations of Trolox from 15 to 300 µmol/mL (y = −0.0026x + 0.7873; R^2^ = 0.9987) and is expressed in µM Trolox equivalent (µM TE/g of sample) ((±)-6-hydroxy-2,5,7,8-tetramethylchroman-2-carboxylic acid, 97%, Sigma, Cotia, SP, Brazil) per gram of extract. The detection limit (DL) was 406.15 µM/mL, and the quantification limit (LQ) was 1230.77 µM/mL, with a standard deviation (σ) of 0.32.

### 2.10. Assessment of Antimicrobial Activity (AM)

The AM was evaluated using the broth microdilution method according to CLSI M07-A11 [28]. The reagents used for this analysis were as follows: Dimethyl sulfoxide (DMSO) was purchased from Dinâmica^®^ Ltda (Indaiatuba, SP, Brazil). Chloramphenicol was purchased from Êxodo Científica^®^ (Sumaré, SP, Brazil). Mueller–Hinton agar (MHA) and Mueller–Hinton broth (MHB) were acquired from Kasvi^®^ (São Paulo, SP, Brazil). Resazurin was obtained from INLAB^®^ (Vila Campestre, SP, Brazil).

The strains used were American Type Culture Collection (ATCC^®^) bacterial strains of *Escherichia coli* (ATCC 25922), *Staphylococcus aureus* (ATCC 25923), and *Pseudomonas aeruginosa* (ATCC 27853), purchased from the Instituto Nacional de Controle de Qualidade em Saúde (INCQS), Fundação Oswaldo Cruz (Fiocruz), Rio de Janeiro, RJ, Brazil. Extracts were dissolved in dimethyl sulfoxide (DMSO, 10% *v*/*v*) and diluted in Mueller–Hinton broth (MHB). Aliquots (100 µL) were transferred to 96-well microplates and subjected to two-fold serial dilutions to obtain final concentrations ranging from 20 to 0.15 mg/mL. Chloramphenicol (1 mg/mL) was used as the positive control, while a solvent control containing the same final concentration of DMSO was included to verify the absence of solvent interference.

Bacterial suspensions were adjusted to a 0.5 McFarland standard (1.5 × 10^8^ CFU/mL) and diluted in MHB to obtain a final inoculum of approximately 5 × 10^5^ CFU/mL in the wells. After the addition of 100 µL of inoculum to each well, the microplates were incubated at 35 ± 2 °C for 24 h in an oven (TLK 48 DeLeo^®^, Porto Alegre, RS, Brazil). Bacterial growth was measured in a spectrophotometer (BK-EL10C, OLABO^®^, Jinan, Shandong, China) at 625 nm, and the inhibition growth percentage (*I*) was calculated according to Equation (1):

(1) $$I\left(\%\right)=\frac{\left({ABS}_{control}-({ABS}_{sample}-{ABS}_{blank})\right)}{{ABS}_{control}}\;\times \;100$$

where *ABS_sample_* is the absorbance of the extract with inoculum after 24 h incubation, *ABS_blank_* is the absorbance of the extract without inoculum, and *ABS_control_* is the absorbance of the inoculum without the extract.

The preliminary antibacterial screening was performed to determine the growth inhibition percentage. For concentrations where visible inhibition was observed via the resazurin assay (0.01% *w*/*v*), the results are expressed as a percentage of inhibition relative to the control. The minimum inhibitory concentration (MIC) was defined as the lowest concentration capable of completely preventing the color change in the indicator [21]. Aliquots (10 µL) from wells showing no visible growth were plated onto Mueller–Hinton agar (MHA) and incubated at 35 ± 2 °C for 24 h. The minimum bactericidal concentration (MBC) was defined as the lowest concentration that resulted in no visible colony formation.

### 2.11. Statistical Analysis

The results were expressed as the mean and standard deviation by quadruplicates for antioxidant and antimicrobial analysis, and the others were used in triplicate. Continuous variables were compared using the two-sample *t*-test or three-sample analysis of variance (ANOVA). When ANOVA revealed a significant difference, a parametric or nonparametric test for mean differences was used, depending on the results of the Shapiro–Wilk normality test. Spearman’s correlation analysis was also performed on the antimicrobial inhibition data, relating it to other important parameters. The significance level established for all statistical tests was *p* = 0.05. The analyses were performed using RStudio software version 4.4.2.

### 2.12. Methodological Description of Spearman’s Correlation

Spearman’s p rank correlation coefficient (Equation (2)) is a non-parametric measure used to determine the magnitude and nature of the monotonic relationship between two continuous or ranked variables [29]. Contrary to traditional parametric methods, Spearman’s coefficient is computed by converting raw values into ranks. It is defined as the difference between the ranks of each value in relation to the number of observations using the formula below:

(2) $$\rho =1-\frac{\sum d_{i}^{2}}{n(n^{2}-1)}$$

where *d_i_* stands for the difference between ranks of the observed values, while n denotes the total number of observations. The coefficient falls between −1 and +1, with values near either extreme implying a perfect monotonic relationship, negative or positive. Due to its lack of restrictive assumptions about the type of distribution, Spearman’s has been found to be suitable for testing the monotonicity of responses of biological systems in the presence of incrementing levels of Alcalase hydrolysates.

## 3. Results

### 3.1. Centesimal Composition

Table 1 shows the centesimal composition of *A. murumuru* biomass, with acid and neutral detergent fibers as the predominant components, representing approximately 64% of the total biomass composition [18].

The lipid content is relatively high (~18%). Proteins represent (~9%) a relevant value for the subsequent production of protein hydrolysate rich in bioactive peptides, while the moisture content is low (~6.5%), favoring material stability during storage.

Finally, the ash content is ~2%, reflecting the presence of minerals, although with considerable variation among samples. This composition reveals the potential of the biomass for both nutritional applications and the extraction of biotechnologically relevant compounds, such as the protein fraction for the production of hydrolysates.

### 3.2. Physicochemical Effects on Protein Solubility, Protein Extraction, and Enzymatic Hydrolysis

**Figure 1:**
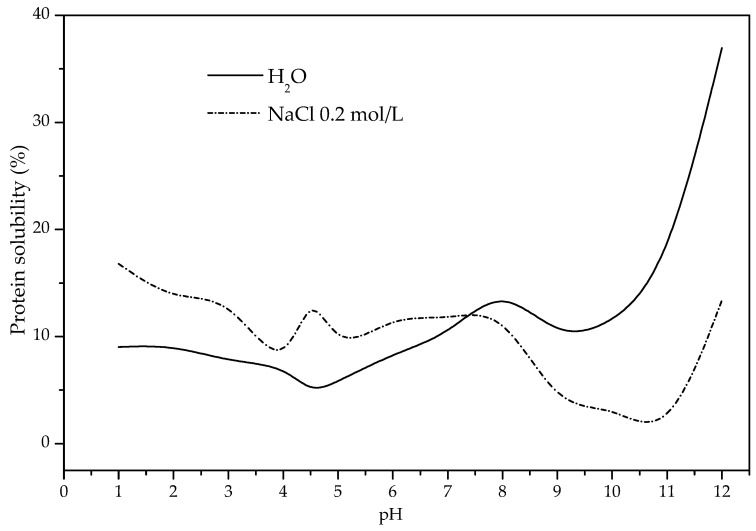
pH-dependent protein solubility profile of defatted *Astrocaryum murumuru* residual biomass. Solubility was determined in distilled water (straight line) and in 0.2 mol/L NaCl solution (dashed line) across a pH range of 1–12. Values represent the mean ± standard deviation (n = 3).

In Figure 1, a characteristic behavior is observed, with pH extremes favoring protein solubility; notably, a pH of 12.0 exhibits the greatest solubility. Solubility generally tends to be greater at alkaline pH values in the range of 8.0 to 12.0 and at acidic pH values, with a minimum in the pH range of 4 to 5, with the minimum being 4.5, corresponding to the isoelectric point (Ip) of this protein.

Table 2 presents the yield and protein content obtained from the extraction of concentrates from *A. murumuru* biomass under different pH and solvent-to-solid ratio conditions. The highest protein yield was achieved at pH 11.0 and a 1:10 (*w*/*v*) ratio, reaching 8.85%, although the total protein content in this condition was comparatively lower (40.79%).

At pH 10.0, yields were lower (4.34–4.90%), but the extracted protein fractions showed higher protein content (47.54–51.03%), particularly at the 1:20 ratio. Similarly, at pH 11.0 with a 1:20 ratio, the yield decreased to 5.92%, while the protein content increased to 50.39%. These results suggest that both the pH and extraction ratio strongly influence recovery efficiency and purity, with pH 11.0 favoring higher yields and pH 10.0 favoring higher protein concentration in the extracts.

The highest yield was less than 9%, observed at pH 11 in a 1:10 ratio. At higher pHs, the protein acquires negative charges and increases its repulsion, increasing its solubility [30]. Considering the low amount of protein available in the biomass, the yield of approximately 9% represents a mass of less than 1 g of protein in 100 g of biomass. This value demonstrates the need to apply other strategies in the extraction step to enable the possible use of the biomass.

#### SDS-PAGE Electrophoresis

**Figure 2:**
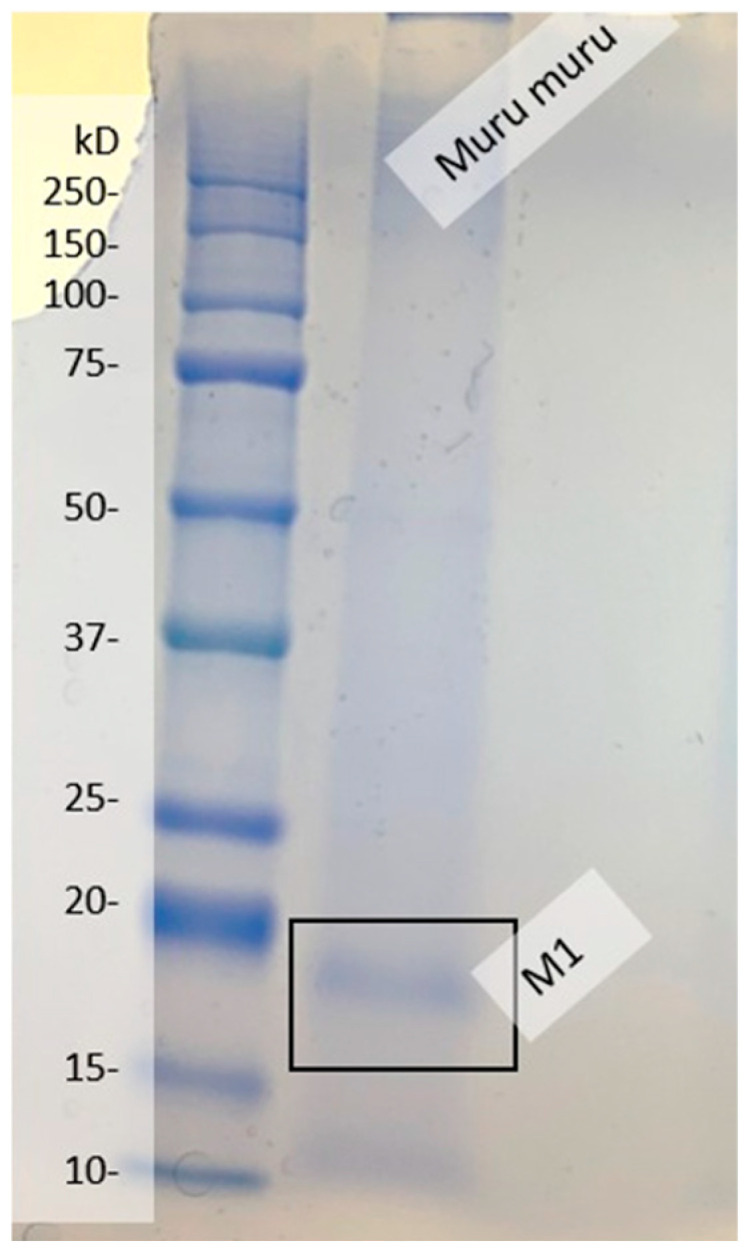
SDS-PAGE analysis (12.5% polyacrylamide gel under reducing conditions, stained with Coomassie blue) of the protein concentrate obtained from the *A. murumuru* biomass under optimized conditions (pH 10.0, 1:20 *w*/*v*). Left line: Molecular weight markers (kDa). Right line: Protein concentrate sample. The square indicates the major protein band at approximately 18 kDa.

Figure 2 shows the electrophoretic profile of the concentrated protein from *A. murumuru* biomass after protein extraction.

The characterization of proteins extracted from *Astrocaryum murumuru* biomass is still limited, with only one evident band at ~18 kDa observed in the analyzed batch. This finding may suggest the presence of prolamins. Still, its confirmation requires additional precise identification through proteomic analyses, which enable the elucidation of the composition and potential functions of these molecules.

### 3.3. Enzymatic Hydrolysis

**Figure 3:**
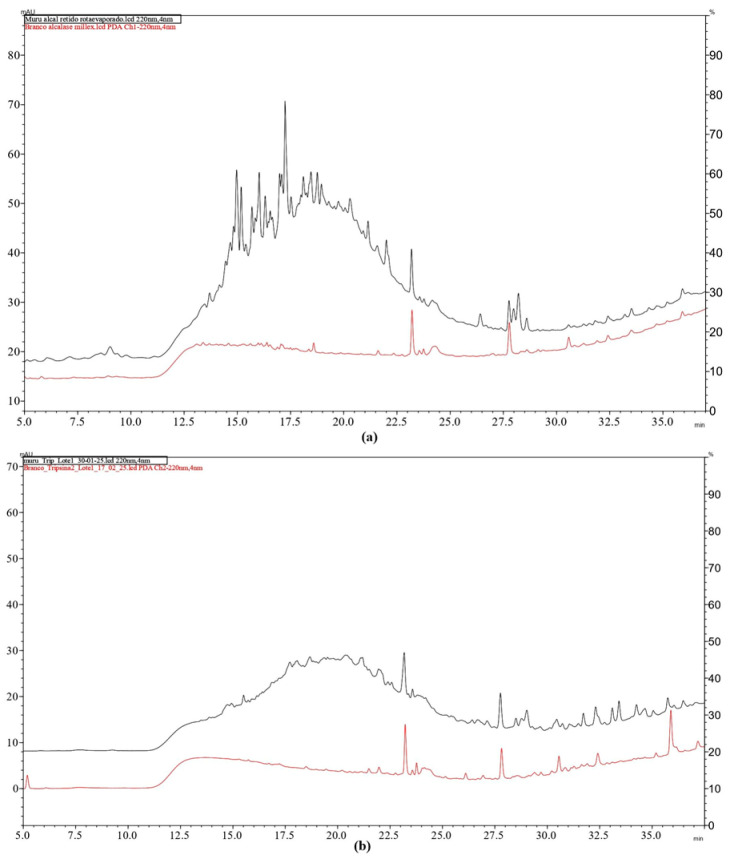
Reversed-phase high-performance liquid chromatography (RP-HPLC) profiles of *Astrocaryum murumuru* protein hydrolysates. Chromatograms show absorbance at 220 nm for hydrolysates generated using (**a**) Alcalase and (**b**) trypsin. Black lines represent the hydrolysates and red lines represent corresponding enzyme blanks (autolysis controls).

Figure 3a,b show the *A. murumuru* protein hydrolysate chromatographic profile with Alcalase and trypsin enzymes, respectively. In both chromatograms, peptide peaks are present in both hydrolysates.

Hydrolysis using Alcalase (Figure 3a) released more peptides at the start of the gradient, presenting three sets of peaks at the beginning of the separation, followed by less intense peaks around 20 min RT. It is also possible to observe the presence of three low-intensity and lower-polarity peaks at retention times between 28 and 29 min.

On the other hand, when the trypsin is used (Figure 3b), peptides are detected at the end of the gradient, with evident peaks at retention times of 29–36 min.

### 3.4. MALDI-TOF Spectrometry of In-Solution Digestions of Extracted Protein

**Figure 4:**
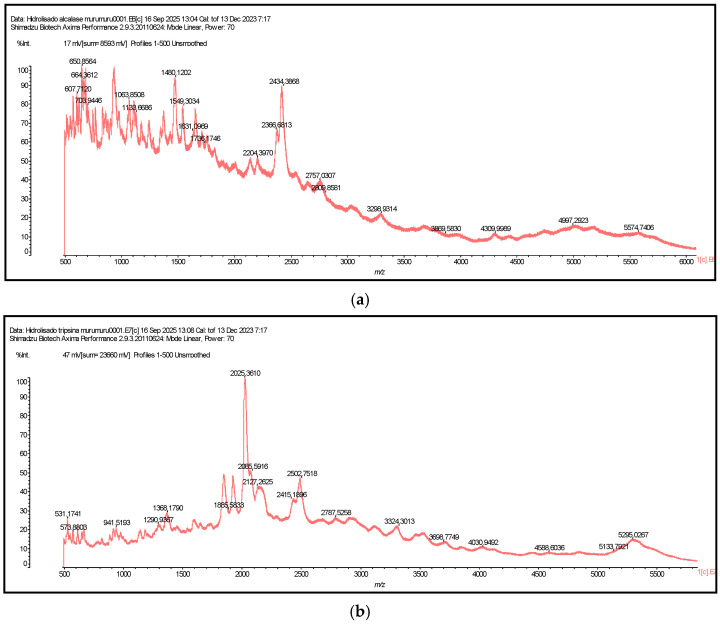
MALDI-TOF mass spectrometry profiles of *Astrocaryum murumuru* protein hydrolysates. Spectra were acquired in positive linear mode using α-cyano-4-hydroxycinnamic acid matrix: (**a**) shows the peptide profile generated by Alcalase hydrolysis, and (**b**) shows the peptide profile generated by trypsin hydrolysis. Major detected peptide ions range from approximately *m*/*z* 500 to 5000.

Figure 4 presents the MALDI-TOF mass spectrometry profiles of the peptides derived from the hydrolysis with Alcalase (Figure 4a) and trypsin (Figure 4b) from the *A. murumuru* protein hydrolysates.

Figure 4a shows the mass spectrum of *A. murumuru* protein hydrolysate digested with Alcalase. For this enzyme, ions are observed in the mass range between ~600 and ~2500 *m*/*z*. Figure 4b shows the mass spectrum of *A. murumuru* protein hydrolysate digested with the trypsin enzyme. A mass range between ~500 and ~5000 *m*/*z* can be observed.

### 3.5. Analysis of TPC, AA, and AM

**Figure 5:**
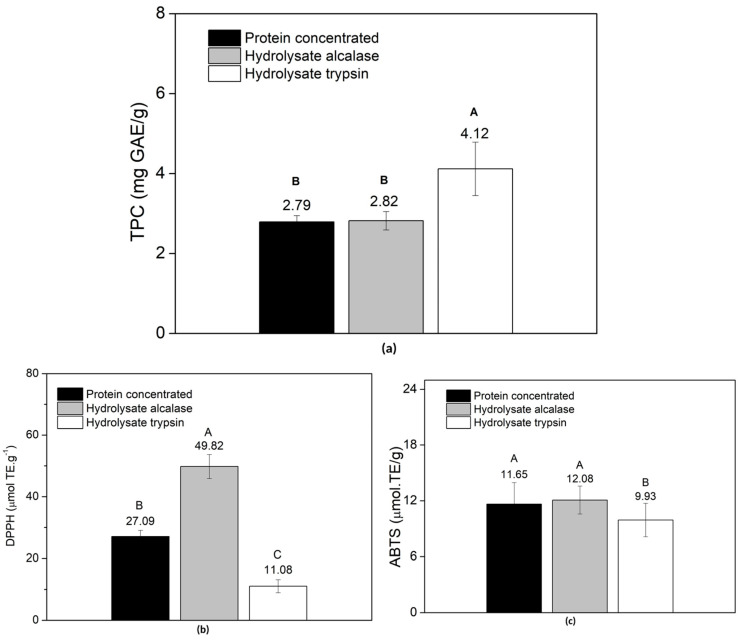
Bioactivity profile of *Astrocaryum murumuru* protein concentrate (PC) and its enzymatic hydrolysates (Alcalase, trypsin): (**a**) total phenolic content (TPC) expressed as mg gallic acid equivalents (GAE)/g sample, (**b**) antioxidant activity determined by DPPH radical scavenging assay, expressed as µmol Trolox equivalents (TE)/g sample, and (**c**) antioxidant activity determined by ABTS radical scavenging assay, expressed as µmol Trolox Equivalents (TE)/g sample. Columns represent mean ± standard deviation (n = 3). Different letters within each panel indicate statistically significant differences between samples according to Tukey’s test (*p <* 0.05).

The results in Figure 5 highlight the TPC and AA by DPPH and ABTS methods for concentrate and protein hydrolysates from *A. murumuru*.

Figure 5a shows that the TPC is significantly higher in the trypsin hydrolyzed fraction with 4.12 mg GAE/g. The protein concentrate and Alcalase hydrolyzed fractions did not show significant differences, with contents of 2.79 and 2.82 mg GAE/g, respectively.

In Figure 5b, the Alcalase hydrolysate showed the highest DPPH radical scavenging activity (49.82 µmol TE/g), surpassing both the protein concentrated (27.9 µmol TE/g) and the trypsin hydrolysate (11.8 µmol TE/g).

For AA by the ABTS method (Figure 5c), the protein concentrate (11.65 µmol TE/g) and the Alcalase hydrolysate (12.08 µmol TE/g) displayed similar and significantly higher values than the trypsin hydrolysate (9.23 µmol TE/g). These findings indicate that enzymatic hydrolysis, particularly with Alcalase, enhances AA in the DPPH assay, while the ABTS results suggest comparable activity between the protein concentrate and the Alcalase hydrolysate, with trypsin hydrolysate and the higher TPC presented being less effective on antioxidant activity.

The AM was assessed by measuring absorbance and performing the resazurin assay, a colorimetric indicator of bacterial metabolic activity. The retention of the blue color indicates a bactericidal effect, whereas a shift to pink signifies bacterial viability. The protein hydrolysates exhibited no inhibitory effect against *E.coli* and *P. aeruginosa*; conversely, they reduced the growth of *S. aureus* with a minimum inhibitory concentration (MIC) of 2.5 mg/mL (Table 3).

Table 3 presents the growth inhibitory activity of *A. murumuru* biomass protein hydrolysates against *S. aureus* across various concentrations. Even though the hydrolysates showed ineffectiveness against *E. coli* and *P. aeruginosa*, a significant concentration-dependent inhibition was observed in *S. aureus* growth. It should be noted that growth inhibition above 90% was observed by using the highest concentrations used in the test for both Alcalase and trypsin hydrolysates. Therefore, the MIC of the bacteria was determined as 2.5 mg/mL because both hydrolysates produced about 92% inhibition at the highest concentration. When comparing the two hydrolysates, it can be stated that trypsin hydrolysate provided 99.85 ± 1.19% growth inhibition while Alcalase hydrolysate provided 93.76 ± 7.06%. Nevertheless, the hydrolysates were not as effective as the chloramphenicol reference substance, which completely inhibited *S. aureus* growth at the lowest concentration (1 mg/mL). Thus, the two hydrolysates were found to inhibit bacteria only bacteriologically, while chloramphenicol inhibited the bacteria both bacteriologically and bactericidally.

The Spearman rank correlation (ρ = 0.8167) is a non-parametric statistical tool used to determine the degree and nature of the monotonic relationship between two variables. While Pearson’s correlation r can only determine linear correlations and is based on a normal distribution of the data (the bell curve), Spearman’s correlation (ρ) does not make any assumptions regarding the distribution of the data set. Instead, it uses ranking [31].

**Figure 6:**
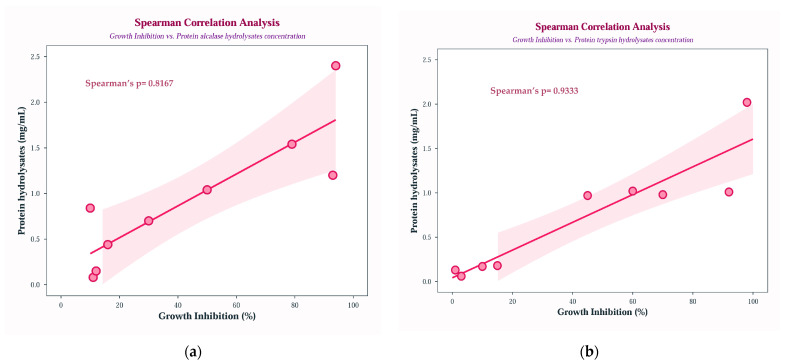
*Spearman’s* correlation analysis between the protein content (g/100 g) of *A. murumuru* hydrolysates and their inhibitory activity (%) against *Staphylococcus aureus:* (**a**) correlation for Alcalase hydrolysate (ρ = 0.8167) and (**b**) correlation for trypsin hydrolysate (ρ = 0.9333). Protein content was determined at each concentration tested in the antimicrobial assay.

*Spearman’s* correlation (ρ) (Figure 6) was performed by relating the protein content present in the hydrolysates, quantified by [24], where the values were 47.74 ± 1.37 and 40.26 ± 4.05 g/100 g for the enzymes Alcalase and trypsin, respectively.

The amount of proteins in each of the concentrations used in the anti-microbial testing was determined and related to the percent inhibition of the *S. aureus* bacteria. Spearman’s correlation coefficients obtained were ρ = 0.8167 (Figure 6a) for Alcalase and ρ = 0.9333 (Figure 6b) for trypsin. The results clearly show that there exists a high positive correlation between the protein concentration in the hydrolysates and the ability to inhibit the growth of *S. aureus* bacteria.

## 4. Discussion

According to Teófilo et al. [32], the moisture content for *A. murumuru* biomass was approximately 5.9%, which is close to the result of the present study, which was 6.46% (Table 1). Therefore, the moisture value of a sample is directly related to the conservation of the food, as it can influence deterioration by microorganisms.

The majority fraction of biomass for the same species is represented by acid and neutral detergent soluble fibers and includes the lignin and cellulose portions, proving to be also an alternative for research aimed at biodegradable materials involving polysaccharides, which can be used in animal feed as a component of silage [18].

Table 1 also reveals that the biomass contains 8.85% protein, a reasonable value since it is a residue. Studies carried out using residues from the agroindustry, such as guava seeds, reported a protein value of 8.43% [33]; however, the study in question was based on residual material from the oil extraction industry. Because it is an agro-industrial residue, the protein fraction present in the biomass becomes an object of exploration because it contains a nutrient that can be used in biotechnology to generate peptides that exert functionalities and bioactivities physicochemical and physiological.

The values found for ash (1.95%) reveal the presence of mineral salts in the biomass. In addition, biomass also has ~17% fat, a considerable value since it is a residue from the oil extraction itself. For biomass of the same species, Serra-Ferreira et al. [18] found 15.1% of fat, demonstrating that this variation of around 2% represents the variation in the extraction process by pressing, since both biomasses were acquired from the same industry. Therefore, this high content is due to the type of extraction carried out by pressing, resulting in a cake with a still high lipid content [34], especially when compared to solvent extraction. The presence of fat interferes with the production of protein isolates, causing emulsions [35]. To prevent this, a solvent degreasing operation had to be performed with hexane in a mass-to-volume ratio of 1:6. This operation, performed on each biomass before the extraction of the proteins, ensures the complete removal of the fatty fraction and prevents the creation of emulsions during the protein extraction process.

In order to explore the protein fraction of biomass to obtain protein hydrolysates, it is important to know the solubility behavior of the protein to define the extraction point and the Ip of this protein. pH influences protein solubility, minimal at the Ip and maximal at higher pH [36]. In an acidic medium, solubility increases, but can decrease at very low pH due to denaturation [19]. Adding NaCl can increase solubility at low concentrations, salting in and reducing it at high concentrations, salting out, and promoting molecular aggregation [37]. Solubility increased from pH 1.0 to 7.0 and decreased from 7.0 to 12.0.

Proteins are being used more extensively in the food industry due to their functional properties, such as emulsification and gel formation, which are influenced by pH, salinity, and processing. Studies are needed to understand the behavior of protein concentrates and isolates. There is no data on the solubility of *A. murumuru* biomass protein.

For *A. murumuru* biomass, protein solubility was higher in alkaline pHs, and the lowest solubility point, i.e., the Ip, is at pH 4.5 (Figure 1). An addition of 0.2 mol/L NaCl caused a slight increase in solubility in the acidic region to the neutral region, with a salting-in effect; however, it was not significant enough to be greater than the water solubility observed in alkaline pHs. Between pH 7.5 and pH 12, the salting-out effect was observed, i.e., protein solubility was reduced at these pHs. It was also observed that the addition of salt to alkaline pHs causes at least protein solubility in the pH 10.5 to 11 region.

Based on the results obtained from Figure 1, conditions were defined for protein extraction, and the yield and protein content of the protein extraction results are in Table 2.

From Table 2, the protein content for both extraction conditions was below 52%; therefore, they are not characterized as a protein isolate (PI). PIs are typically defined as having a protein content of at least 90% [36]. However, the methodology normally employed to obtain protein isolates, alkaline solubilization followed by isoelectric precipitation, used in the extraction of *A. murumuru* protein, promoted a protein concentration of approximately six times the protein content found in the defatted biomass.

The higher content protein was obtained when the 1:20 ratio was used at pH levels of 10 and 11, with protein concentrations of 51.03% and 50.39%, respectively. However, the highest yield was observed at pH 11.0, with 8.85% of the sample.

A plausible reason for the difficulty in obtaining PI from *A. murumuru* biomass may be the presence of phenolic compounds in its composition. In another study conducted by our group, we found significant levels of phenolic compounds (9.92 mg GAE/g), flavonoids (0.38 mg QE/g), and tannins (4.03 mg TA/g) in an *A. murumuru* biomass [8].

Furthermore, the high acid and neutral detergent fiber content in the biomass hinders access to the protein fraction, leading to low extraction yields, which demonstrates the need to improve processes for obtaining the protein isolate from this biomass.

Neji et al. [38] used data analysis to optimize soy protein extraction; among the important parameters reported by the authors are the temperature and pH of isoelectric precipitation and extraction. In this work, the effect of temperature on protein extraction was not explored. In addition to pH and temperature, extraction time is also identified as an optimization factor in protein extraction [39].

Considering the composition of biomass, according to our results, a mass-volume ratio of 1:20 for 60 min and pH 10.0 were selected as better conditions for obtaining the concentrated protein from the *A. murumuru* biomass.

Protein characterization was performed by electrophoresis, and the presence of a more evident protein band (~18 kDa) is indicated in Figure 2, justifying the possibility of obtaining peptides after enzymatic hydrolysis.

From the samples of concentrated proteins, it was possible to obtain protein hydrolysates using enzymes widely used in food biotechnology to generate bioactive peptides. Given that this is an initial investigation into the biotechnological exploitation of this biomass, coupled with its low protein yield and concerns about environmental impact, hydrolysis times previously established in the literature were adopted to ensure efficient protein cleavage. The protein hydrolysates were evaluated by liquid chromatography (HPLC) and mass spectrometry (MALDI-TOF). It must also be pointed out that the precise regulation of the level of hydrolysis and the exact determination of the structure of the peptides formed are planned for subsequent stages of the study in order to obtain a better understanding of protein structure and function.

The chromatographic profiles presented in Figure 3 show that the peptide profiles were different for each enzyme type (Figure 3a for Alcalase and Figure 3b for trypsin), with more nonpolar peptides in tryptic hydrolysis and a larger set of more polar peptides in hydrolysis with Alcalase.

The enzyme trypsin has cleavage specificity at lysine and arginine residues [30], and the low concentration of these amino acids in the protein may be indicative of the smaller number of peaks found in Figure 3b. On the other hand, the Alcalase enzyme has greater specificity, cleaving bonds in the amino acids glutamine, methionine, leucine, tyrosine, and glycine at the P_1_ position, preferentially with the presence of a hydrophobic residue at the P_2_′ or P_3_′ position, generating larger quantities of shorter-chain peptides with high hydrophobicity [40].

The MALDI-TOF mass spectrometric analyses (Figure 4a,b) confirmed the efficiency of enzymatic hydrolysis of *A. murumuru*. This finding is further supported by the RP-HPLC chromatograms (Figure 3a,b). Together, these analytical approaches demonstrate not only the successful generation of peptides but also highlight their structural diversity, which may be directly related to the antioxidant and antimicrobial activities observed.

Both hydrolysates and *A. murumuru* concentrated protein had their TPC determined. Figure 5a reveals that the number of TPC was significantly concentrated in the trypsin protein hydrolysate when compared to the concentrated protein itself. According to [41], the formation of protein–phenolic complexes tends to occur preferentially through covalent bonds via spontaneous oxidation of some phenolics and through non-covalent bonds, such as hydrophobic, hydrogen, and ionic bonds. The same authors also state that the preferential binding site has been identified for the amino acid lysine and the thiol groups of cysteine.

The AA of protein hydrolysates was evaluated using the DPPH and ABTS methods (Figure 5b,c), which measure the capacity of different compounds to scavenge free radicals.

The results showed that the hydrolysates presented AA, indicating the presence of peptides and/or phenolic compounds capable of donating electrons or hydrogen to stabilize free radicals [42]. Generally, the hydrolysates obtained with Alcalase presented greater antioxidant activity than those obtained with trypsin, although this trend was not uniform for all biomasses. Considering that the hydrolysate obtained with trypsin presented the highest number of phenolic compounds, this effect cannot be attributed to this fraction. Figure 5b,c reveal that the fraction that presents the highest amount of phenolics was the fraction that presented the lowest AA, suggesting that the peptides present in the hydrolysate with Alcalase were more effective in this bioactivity. This could be due to the specificity of enzymes, as they break down proteins at different locations, producing peptides with different amino acid sequences, and hence different antioxidant potentials.

Karamac et al. [43] found similar behavior for flaxseed protein hydrolysates, with Alcalase hydrolysates showing higher AA than that obtained by trypsin using the ABTS method, finding values of approximately 0.21 and 0.20 µmol—TE/g, respectively. However, in the current work, the AA values were 12.08 and 9.93 µmol TE/g for Alcalase and trypsin hydrolysate, respectively, about 57 times higher for Alcalase and 49 times for trypsin, demonstrating the presence of peptides with a greater effect for *A. murumuru.*

On the other hand, when compared with OKARA, a soy-based residue from the processing of soy milk and tofu, higher AA values were observed, dependent on the degree of hydrolysis, presenting an AA of 300 µmol TE/g after 5 min of hydrolysis, reaching 700 µmol TE/g after 3 h of hydrolysis [44]. However, the authors used the FRAP method (ferric reducing antioxidant power).

Regarding the growth inhibition results, it is observed that at intermediate and lower concentrations, differences between the two enzymes became more evident. The growth inhibition potency of trypsin hydrolysate was found to be relatively high (45.11 ± 0.54%) at 1.75 mg/mL compared to that of Alcalase hydrolysate, which decreased dramatically to 9.85 ± 3.97%. The growth inhibition potency of both enzymes was low at concentrations of 0.312 mg/mL and 0.156 mg/mL; it was below 12%, but the Alcalase enzyme demonstrated a growth inhibition potency that was a little bit higher than the trypsin enzyme. At the level of 5 mg/mL, the trypsin enzyme inhibited over 99% of the microbial growth, while the Alcalase enzyme inhibited 93%.

Wang et al. [45] used an alternative protein, derived from *Moringa oleifera* seeds, and obtained a protein hydrolysate that showed an inhibition rate below 30% at 5 mg/mL concentration, and below 90% using 20 mg/mL. However, fractionating the hydrolysate found a high inhibition rate in fraction three, which contained five peptides, one of them termed MOp3, which showed a minimum inhibition concentration for *S. aureus* at a 2 mg/mL concentration. This work opens up perspectives for further investigation into murumuru hydrolysate through individual testing of fractions and respective peptide identification.

A recent evaluation of the AM of the hydroalcoholic extract of *A. murumuru* biomass [8] revealed similarities in the spectrum of action when compared to the findings of this research, but with significant differences in growth inhibitory potency. Both bioproducts demonstrated selective action, reducing the growth of the Gram-positive bacterium *S. aureus*, without showing efficacy against the Gram-negative strains of *E. coli* and *P. aeruginosa*. Furthermore, both the extract and the hydrolysates exhibited a purely bacteriostatic mechanism against *S. aureus*, since neither treatment was able to show bactericidal activity or establish an MBC under the tested conditions. However, while the inhibition promoted by the ethanolic extract ranged from 48.24% (at 1.17 mg/mL) to a limit of 77.78% (at 10 mg/mL), the protein hydrolysates obtained from the same raw material showed considerably superior inhibitory behavior. This superiority can be attributed to the physicochemical nature of the bioactive peptides released during enzymatic hydrolysis. Unlike the complex phytochemical compounds present in crude extracts, antimicrobial peptides (AMPs) often have low molecular weight, amphiphilic character, and a net positive charge (cationic) [46].

Although further studies were not conducted in this study to confirm this claim, the literature suggests mechanisms, such as these structural characteristics, may give the peptides a strong electrostatic attraction to the negatively charged surface of *S. aureus* (rich in teichoic acids in the thick peptidoglycan layer) [47]. This may allow the peptide fractions to interact, permeate, and interfere with the membrane or metabolic processes of the bacteria with much greater efficiency and specificity than the secondary compounds of the extract, resulting in high inhibition rates at reduced concentrations [48].

Most antimicrobial peptides belong to antibacterial, fungicidal, antiviral, and antiparasitic groups. The antibacterial action is attributed to its amphiphilic nature and the presence of highly charged residues in its structure. This arrangement facilitates the attachment and insertion of peptides into the bacterial membrane, creating transmembrane pores that result in permeabilization. The amphipathic nature of antimicrobial peptides is key to this process, as the hydrophobic domains interact directly with the lipid components of the membrane, while the hydrophilic cationic groups interact with the phospholipid groups also present in the membrane [49].

While the literature proposes modes of action such as membrane permeability, further research using SEM and membrane potential techniques is required in order to determine the actual modes of action of these new *A. murumuru* peptides.

The association between the hydrolysate protein composition and its ability to inhibit *S. aureus* growth was quantitatively demonstrated by the Spearman correlation analysis (Figure 6a,b) used in this investigation. The hydrolysate obtained with Alcalase had a Spearman’s correlation coefficient of ρ = 0.8167, whereas the hydrolysate obtained with trypsin had a correlation coefficient of ρ = 0.9333. These results show a high positive correlation between the percentage of bacterial inhibition and the concentration of proteins/peptides.

These results support the theory that bioactive peptides are the primary agents inhibiting *S. aureus* by indicating that the AM increases proportionately with the content of peptides in the hydrolysates. When compared to the peptides produced by Alcalase, the stronger correlation found for the tryptic hydrolysate suggests that the peptides produced by this enzyme have a higher and more inhibitory bacterial growth effectiveness per unit of protein. From a biochemical point of view, this difference can be explained by the specificity of trypsin, which preferentially cleaves peptide bonds involving lysine and arginine, resulting in peptides with a greater cationic character as discussed before.

Furthermore, the absence of activity against *E. coli* and *P. aeruginosa* reinforces that the growth inhibitory activity of the hydrolysates depends not only on the peptide concentration but also on the structural susceptibility of the target microorganism. This occurs because Gram-negative bacteria possess an outer membrane rich in lipopolysaccharides, which acts as a barrier and hinders the penetration of these inhibitory peptides.

Analysis of the inhibition properties of the protein hydrolysates demonstrated a significant and positive correlation between the rise in the amount of bioactive peptides and their inhibitory potential. Because of the non-linear nature and monotone properties of biological reactions, the Spearman correlation method (ρ) was used. By using a statistical model that does not involve strict linear conditions, it was possible to assess the true nature of the relationship between the variables under consideration without losing precision due to the limitations of Spearman’s models, which is important in the context of the biological process being examined, where the behavior of biological samples must be accurately monitored [29].

The inhibition behavior showed different sensitivities with regard to the type of specificity of the enzyme used during the hydrolysis procedure. For the hydrolysate obtained through the use of Alcalase (Figure 6a), a robust monotonic relationship was observed (ρ = 0.8167). Even though there appears to be a tendency for some clustering of values near the MIC range, as depicted on the graph, the model was robust enough to handle biological variation and the subsequent increase in activity seen at higher concentrations. Such a response is typical in a complex peptide matrix in which bioactivity is not controlled by one isolated peptide molecule but by synergy and the distribution of the molar mass profile of the peptides produced during the hydrolysis process.

In contrast, the hydrolysis effect caused by the presence of trypsin (Figure 6b) exhibited a much more significant statistical correlation, being considered very strong (Spearman’s ρ = 0.9333). The improved location of experimental data points within the range of growth inhibition gradient suggests a more predictable and even dose-dependent effect exerted on the bacterial growth by trypsin rather than Alcalase. In terms of biochemistry, such a difference may be explained by the different nature of the enzymes: Alcalase is a nonspecific endopeptidase producing a very heterogeneous population of small peptides, whereas trypsin possesses highly specific cleavage sites (Lys and Arg residues). Such specificity results in greater homogeneity and predictability of molecular profiles; thus, the interaction dynamics with the receptor or membrane destabilization occur according to the established dose–effect relationship. As highlighted by Nascimento et al. [50], the homogeneity of multivariate correlations of biological inhibition is associated with a high level of chemical stability of the active molecules, which explains the high *p*-value observed in the case of tryptic hydrolysis.

## 5. Conclusions

This research represents the first study describing the bioactive properties of peptides isolated from residual biomass of *A. murumuru*, an underutilized Amazonian waste product. Peptide-rich hydrolysates were successfully obtained through the optimized extraction process of proteins and subsequent treatment with selected enzymes—Alcalase and trypsin. One important observation made is the functional difference caused by enzyme application, where Alcalase hydrolysis increased the ability of the samples to scavenge DPPH radicals (49.82 µmol TE/g), which was significantly higher than that exhibited by the non-hydrolyzed sample. The results obtained were compared with the amount of total phenolics present, thus indicating that peptides are responsible for the observed antioxidant activity. As for the trypsin hydrolysis, peptides showed strong antimicrobial activity against *S. aureus* (MIC 2.5 mg/mL) at an almost complete inhibition rate (99.85%) at 5 mg/mL, where the relationship between the antibacterial activity and the amount of peptides was significant (r = 0.8384).

These results indicate that the controlled enzymatic hydrolysis of *A. murumuru* protein can produce hydrolysates with distinct bioactivities, tailored by protease selection. The antioxidant properties associated with Alcalase-derived peptides suggest potential utility as natural preservatives or functional ingredients. Concurrently, the anti-*S. aureus* activity of trypsin-derived peptides warrants further investigation into applications requiring targeted Gram-positive bacterial inhibition, such as in food safety or topical formulations. This work validates *A. murumuru* biomass residue as a valuable substrate for producing functional protein hydrolysates, contributing to sustainable waste valorization within the Amazon bioeconomy framework. Despite having a low protein content in biomass composition, after enzymatic hydrolysis, this material demonstrated antioxidant and antimicrobial activities, suggesting the peptides generated by this protein showed bioactivity, which enhances the potential of exploration to identify peptides of interest. Future studies involving peptide fractionation, sequencing (e.g., LC-MS/MS), and detailed mechanistic investigations are necessary to identify the specific bioactive sequences and fully elucidate their modes of action. Nevertheless, the present findings establish *A. murumuru residue* as a relevant raw material for developing sustainable bioproducts with defined functional properties.

## Figures and Tables

**Table 1:** Proximate composition (% dry weight basis) of the residual biomass from *A. murumuru* oil extraction.

Components	Average ± SD
Moisture	6.46 ± 0.60
Proteins *	8.85 ± 0.17
Lipids	17.82 ± 5.64
Ashes	1.95 ± 2.38
Fibers **	~64

Notes: * FC = 6.25; ** obtained by difference and compared with the literature for the same species biomass [18].

**Table 2:** Effect of pH and solid-to-solvent ratio on the extraction yield and protein content of concentrates obtained from defatted *Astrocaryum murumuru* biomass.

pH Extraction	Ratio (*w*/*v*)	Yield of Concentrate (%) *	Total Protein Content (%)
10.0	1:10	4.34	47.54
10.0	1:20	4.90	51.03
11.0	1:10	8.85	40.79
11.0	1:20	5.92	50.39

* yield percentages were calculated based on the protein content in the sample mass obtained.

**Table 3:** Concentration-dependent growth inhibition of *Astrocaryum murumuru* protein hydrolysates (Alcalase and trypsin) against the growth inhibition of *Staphylococcus aureus* (ATCC 25923).

Concentration (mg/mL)	Growth Inhibition (%)	
	Alcalase Hydrolysate	Trypsin Hydrolysate
5	93.76 ± 7.06 ^b^	99.85 ± 1.19 ^a^
2.5	92.72 ± 3.17 ^a^	92.62 ± 0.64 ^a^
1.7	9.85 ± 3.97 ^b^	45.11 ± 0.54 ^a^
0.3	11.97 ± 4.21 ^a^	1.12 ± 0.49 ^b^
0.1	12.13 ± 1.14 ^a^	3.13 ± 0.78 ^b^

Notes: Averages with different letters on the same line indicate a significant difference using the Mann–Whitney test (*p* < 0.05).

## Data Availability

The original contributions presented in this study are included in the article. Further inquiries can be directed to the corresponding author.

## Abbreviations

The following abbreviations are used in this manuscript:

AA	Antioxidant Activity
ABTS	2,2′-Azino-bis(3-ethylbenzothiazoline-6-sulfonic acid)
AM	Antimicrobial Activity
ANOVA	Analysis of Variance
DL	Detection Limit
DPPH	2,2-Diphenyl-1-picrylhydrazyl
GAE	Gallic Acid Equivalent
Ip	Isoelectric Point
LC-MS	Liquid Chromatography–Mass Spectrometry
LQ	Quantification Limit
MALDI-TOF	Matrix-Assisted Laser Desorption/Ionization Time-of-Flight
MIC	Minimum Inhibitory Concentration
MBC	Minimum bactericidal concentration
PDA	Photodiode Array Detector
PI	Protein Isolate
PC	Protein Concentrate
RP-HPLC	Reverse Phase—High Performance Liquid Chromatography
SDS-PAGE	Sodium Dodecyl-Sulfate Polyacrylamide Gel Electrophoresis
TPC	Total Phenolic Content
